# The PRIAMO study: age- and sex-related relationship between prodromal constipation and disease phenotype in early Parkinson’s disease

**DOI:** 10.1007/s00415-020-10156-3

**Published:** 2020-08-18

**Authors:** Marina Picillo, Raffaele Palladino, Roberto Erro, Rossella Alfano, Carlo Colosimo, Roberto Marconi, Angelo Antonini, Paolo Barone, on behalf of the PRIAMO study group, Angelo Antonini, Carlo Colosimo, Roberto Marconi, Letterio Morgante, D. Benincasa, R. Quatrale, S. Biguzzi, M. Braga, G. Ceravolo, M. Capecci, G. Meco, N. Caravona, R. Scala, F. A. De Falco, G. Pezzoli, D. De Gaspari, E. Bottacchi, M. Di Giovanni, A. Cannas, G. Floris, S. Gallerini, L. Grasso, R. M. Gaglio, G. Gurgone, G. Volpe, S. Zappulla, R. Ceravolo, L. Kiferle, S. Ramat, S. Meoni, A. Pisani, V. Moschella, F. Morgante, R. Savica, F. Pepe, G. Ciccarelli, V. Petretta, R. M. Giglia, M. G. Randisi, F. Iemolo, T. P. Avarello, M. Romeno, G. Santangelo, F. Stocchi, G. Sciortino, V. Sorbello, A. Nicoletti, D. Tiple, G. Fabbrini, A. Bentivoglio, F. E. Pontieri, A. Guidubaldi, R. Muoio, V. Toni, P. Del Dotto, C. Logi, G. Ciacci, M. Ulivelli, M. Perini, S. Lanfranchi, S. Griffini, B. Troianiello, M. Baratti, S. Amidei, D. Consoli, M. Iellamo, T. Cuomo, A. Scaglioni, D. Medici, M. Manfredi, G. Abbruzzese, G. Di Brigida, G. A. Cocco, V. Agnetti, G. Cossu, M. Deriu, M. Abrignani, C. Modica, G. Albani, E. Milan, P. Martinelli, C. Scaglione, M. Mucchiut, S. Zanini, F. Pennisi, P Soliveri, A. Albanese, Pederzoli Massimo, L. Bartolomei, L. Capus, L. Ferigo, R. Marano, V. Nastasi, R. Luciano, L. Maiello, P. Simone, D. Fogli, L. Lopiano, M. Pesare, G. Nordera, E. Pilleri, T. Scaravilli, E. Giaccaglini, C. Alesi, A. Petrone, G. Trianni

**Affiliations:** 1 Department of Medicine, Surgery and Dentistry, Neuroscience Section, Center for Neurodegenerative Diseases (CEMAND), University of Salerno, Salerno, 84131 Italy; 2 Department of Primary Care and Public Health, School of Public Health, Imperial College of London, London, UK; 3 Department of Public Health, School of Medicine, University “Federico II”, Naples, Italy; 4 Department of Neurology, Santa Maria University Hospital, Terni, Italy; 5 Neurology Division, Misericordia Hospital, Grosseto, Italy; 6 Department of Neurosciences (DNS), Padova University, Padua, Italy

**Keywords:** Parkinson, Constipation, Prodromal, Phenotype, Heterogeneity, Sex

## Abstract

**Objectives:**

To explore the impact of sex and age on relationship between prodromal constipation and disease phenotype in Parkinson’s disease at early stages.

**Methods:**

A total of 385 Parkinson’s disease patients from the PRIAMO study were classified according to the presence of prodromal constipation and followed for 24 months. Multivariable mixed-effect models were applied. All analyses were performed separately for sex (64.1% men) and median age (different by sex: 67 years-old in men and 68 years-old in women).

**Results:**

As for sex, prodromal constipation was associated with greater odds of attention/memory complaints and apathy symptoms in women only. As for age, prodromal constipation was associated with lower cognitive and higher apathy scores in older patients only.

**Conclusions:**

Prodromal constipation anticipates lower cognitive performances and more severe apathy since the earliest stages in women and older patients. Sex- and age-related heterogeneity of prodromal markers of Parkinson’s disease may impact disease phenotype.

**Electronic supplementary material:**

The online version of this article (10.1007/s00415-020-10156-3) contains supplementary material, which is available to authorized users.

## Introduction

The Movement Disorder Society (MDS) research criteria for prodromal Parkinson’s disease (PD) provide a methodological framework to estimate the likelihood of developing the disease based on the presence of specific features [1]. Such criteria have been validated in prospective cohort studies as well as in primary care settings [2, 3]. As sex and age may impact the predictive value of single risks and prodromal markers of PD, both such factors need to be considered when developing risk models and tools for earlier detection of the disease [4].

Constipation represents one of the most solid prodromal markers of PD (positive likelihood ratio, LR + = 2.5) [1]. Recent evidence suggests higher odds of developing PD for women and subjects with constipation aged above 65 year-old [4]. However, constipation is a common gastrointestinal disorder in general population with prevalence up to 30% and, similarly to PD, more prevalent in women and elderly [5–7]. Both the presence of gastrointestinal pathological α—synuclein deposits and constipation in prodromal and clinically established PD suggests an integral role of the gut—brain axis for the early pathogenesis of the disease. According to such hypothesis, the synucleinopathy is hypothesized to ascend via the vagal nerve from peripheral neurons of the gastrointestinal tract to the brain [1–4, 8, 9].

To date, it is unknown if in PD the higher prevalence of prodromal constipation may have an impact on disease phenotype and progression. As such, no data is available on the relationship between the presence of prodromal constipation (PC) and disease phenotype at early stage.

By further analyzing prospective data from a subset of patients of the PRIAMO (PaRkinson dIseAse non-MOtor symptoms) study [10, 11] the present work aims to explore the relationship between the presence of prodromal constipation and disease phenotype in the early stages considering the impact of sex and age.

## Patients and methods

### Patients and assessments

This study involves a subgroup of patients from the PRIAMO study. The PRIAMO study is a large Italian multicentre observational study designed to assess the prevalence and evolution of NMS in patients affected by different parkinsonian syndromes and including a cross sectional and a longitudinal prospective 24-month phase [12–14].

Study methods and patient baseline features have been extensively described elsewhere [10, 11]. Each patient underwent a baseline (T1) and two follow-up visits, at 12 months (± 4) after the baseline visit (T2) and 9-16 months after the first follow-up visit (T3), respectively. Diagnosis of idiopathic PD was based on Gelb et al., criteria. Enrolled patients were administered a semistructured interview exploring 12 NMS domains (gastrointestinal, urinary, pain, cardiovascular, sleep, fatigue, apathy, attention/memory complaints, skin, psychiatric, respiratory and other symptoms including smell and taste impairment, diplopia, weight change), each one including 2–10 specific questions with dichotomous (yes/no) answers (see supplemental material online) [10, 11].

In addition, only patients reporting gastrointestinal dysfunction in the semistructured interview were asked if they had developed constipation before the onset of motor symptoms (yes/no). All the clinicians involved were expert in movement disorders and participated in a training session before starting enrolment for the PRIAMO study.

The Unified Parkinson’s Disease Rating Scale-part III (UPDRS-III) was used to evaluate motor disability, while cognitive abilities were investigated with the Mini-Mental State Examination (MMSE). Depressive symptoms were evaluated with the Hamilton Depression scale (HAM). HR-Qol was investigated using the 39-item Parkinson’s disease questionnaire (PDQ-39) and the EuroQol visual analogue scale (EQ-VAS).

For this subanalysis, only patients with early stage PD [namely, Hoehn and Yahr (H&Y) stage ≤ 2] at baseline, with no missing answers to the gateway question on PC, and with at least one follow-up assessment were considered. The study was approved by the ethics committees of the participating centers and all patients provided written informed consent.

### Statistical analysis

Of 1142 PD patients enrolled in the PRIAMO study, 385 (247 men and 138 women) PD patients had H&Y stage ≤ 2 at baseline and with no missing answers to the gateway question on PC and at least one follow-up assessment, and, thus, were eligible for the present analysis. Data at follow-up were available for 322 patients (of whom 209 men) at T2 and 297 patients (of whom 193 men) at T3.

Given the aim of the present work, all the analyses were performed separately for sex (64.1% men) and median age by sex (i.e., 67 years in men and 68 years in women).

After checking for normal distribution of variables, comparisons between groups were performed using *χ*^2^, *t* test and analysis of variance (ANOVA) as appropriate. Accordingly, descriptive statistics are presented as proportions, or mean and standard deviation (SD). To test the association between PC (the independent variable) and NMS, L-dopa treatment, UPDRS-III, MMSE, HAM, PDQ-39 and EQ-VAS (modelled as the dependent variables) over time, we used multivariate mixed effect logistic (for categorical variables: NMS and L-dopa treatment) and linear (for continuous variables: UPDRS-III, MMSE, HAM, PDQ-39 and EQ-VAS) regression models fitted with random patients intercept and adjusted for disease duration, time point and, according to the specific model, patients’ age or sex. A part from the separate analysis on the impact of age considered as a binomial variable, age was included as a continuous variable (refer to supplemental material for detailed results). Considering the possible role of both age and sex as effect modifiers in association with our study outcomes, in our model building strategy we also tested for an interaction term between age and sex. However, the interaction term was not significant. Hence, the interaction was excluded from the model to reduce degrees of freedom.

Associations were considered significant if *p*-values were lower than 0.05. Coefficients are presented as adjusted estimates (continuous dependent variables) or odds ratios (categorical dependent variables) along with 95% confidence intervals (95% CI).

Statistical analyses were performed using the lme4 package in the R software (version 3.4.1).

## Results

Detailed demographic and clinical features of the included cohort by sex at T1, T2 and T3 are displayed in Supplemental material online.

### Relationship with sex

Prevalence of PC was 34% in men and 45.6% in women (*p* = 0.024). In women, PC was associated with greater odd of endorsing attention/memory complaints (OR: 4.35, 95%CI: 1.13–16.72, *p* = 0.032) and apathy (OR: 3.49, 95%CI: 1.05–11.59, *p* = 0.041) domain, lower probability of being treated with levodopa (OR: 0.12, 95%CI: 0.02–0.76, *p* = 0.025) and a trend towards significance for lower MMSE scores (coeff.: − 1.45, 95%CI: − 2.94 to 0.03, *p* = 0.055). Conversely, in men PC was only associated with lower probability of complaining of other NMS (OR: 0.35, 95%CI: 0.13–0.94, *p* = 0.037) (Table 1).

**Table 1:** Association between prodromal constipation and motor and non-motor symptoms in early PD according to sex and median age

	Men				Women				Younger patients				Older patients			
Continuous variables	Estimate	LCI	UCI	*p*	Estimate	LCI	UCI	*p*	Estimate	LCI	UCI	*p*	Estimate	LCI	UCI	*p*
UPDRS-III	− 1.58	− 3.96	0.80	0.192	0.62	− 2.52	3.76	0.699	− 0.91	− 3.56	1.74	0.501	1.28	− 2.23	4.80	0.475
MMSE	− 0.71	− 1.67	0.25	0.147	− 1.45	− 2.94	0.03	0.055	− 0.68	− 1.81	0.45	0.240	− 2.48	− 4.00	− 0.96	**0.001**
EQ-VAS	− 2.46	− 6.60	1.69	0.245	3.26	− 2.53	9.05	0.269	0.35	− 4.36	5.07	0.884	− 5.43	− 11.71	0.84	0.090
PDQ-39	9.60	− 16.96	36.16	0.479	− 0.25	− 44.04	43.54	0.991	3.93	− 27.21	35.08	0.804	33.52	− 8.37	75.43	0.117
HAM	0.17	− 0.81	1.14	0.738	1.39	− 0.16	2.94	0.079	0.07	− 1.09	1.25	0.894	2.07	0.51	3.62	**0.009**

Categorical variables	OR	LCI	UCI	*p*	OR	LCI	UCI	*p*	OR	LCI	UCI	*p*	OR	LCI	UCI	*p*
L-dopa treatment	0.86	0.25	2.91	0.806	0.12	0.02	0.76	**0.025**	0.46	0.12	1.77	0.263	11.58	1.85	72.40	**0.009**
Urinary	1.26	0.52	3.05	0.601	1.78	0.41	7.68	0.437	2.52	0.85	7.42	0.093	1.52	0.36	6.37	0.562
Pain	1.08	0.48	2.40	0.859	0.39	0.13	1.18	0.096	1.29	0.52	3.18	0.574	1.24	0.37	4.11	0.722
Cardiovascular	2.09	0.71	6.09	0.179	0.89	0.31	2.56	0.832	0.84	0.27	2.61	0.773	1.64	0.39	6.80	0.494
Sleep	0.55	0.25	1.25	0.154	1.74	0.54	5.62	0.354	1.13	0.45	2.85	0.790	1.82	0.52	6.28	0.344
Fatigue	0.86	0.37	1.96	0.713	0.54	0.17	1.68	0.287	0.73	0.29	1.85	0.519	2.23	0.64	7.75	0.207
Apathy	1.51	0.57	4.00	0.406	3.49	1.05	11.59	**0.041**	0.69	0.24	1.99	0.500	7.34	1.79	30.07	**0.006**
Attention/memory	1.63	0.72	3.71	0.245	4.35	1.13	16.72	**0.032**	1.80	0.69	4.69	0.227	3.46	0.96	12.49	0.057
Skin	0.82	0.30	2.23	0.701	0.69	0.17	2.85	0.611	1.11	0.38	3.20	0.847	0.26	0.05	1.26	0.095
Psychiatric	1.46	0.67	3.19	0.336	1.40	0.46	4.26	0.554	1.01	0.42	2.45	0.972	1.90	0.58	6.21	0.283
Respiratory	1.46	0.65	3.29	0.356	0.39	0.09	1.63	0.196	0.48	0.16	1.37	0.172	0.70	0.19	2.54	0.590
Other symptoms	0.35	0.13	0.94	**0.037**	0.75	0.15	3.81	0.730	1.03	0.32	3.32	0.956	1.00	0.21	4.83	0.991

Coefficients are presented as adjusted estimates (for continuous dependent variables) or odds ratios (for categorical dependent variables) along with 95% confidence intervals (95%CI) and were estimated using linear (for continuous dependent variables) and logistic (for categorical dependent variables) mixed effects regression models, adjusting for age, disease duration and time point, introducing a random effect for participant. Significant results are highlighted in bold

*EQ-VAS* EuroQol visual analogue scale, *HAM* the Hamilton depression scale, *LCI* lower 95% confidence interval, *MMSE* Mini-mental state examination, *OR* odds ratio, *PD* Parkinson’s disease, *PDQ-39* 39-item Parkinson’s disease questionnaire, *UCI* upper 95% confidence interval, *UPDRS-III* Unified Parkinson’s disease rating scale part III

### Relationship with age

**Fig. 1:**
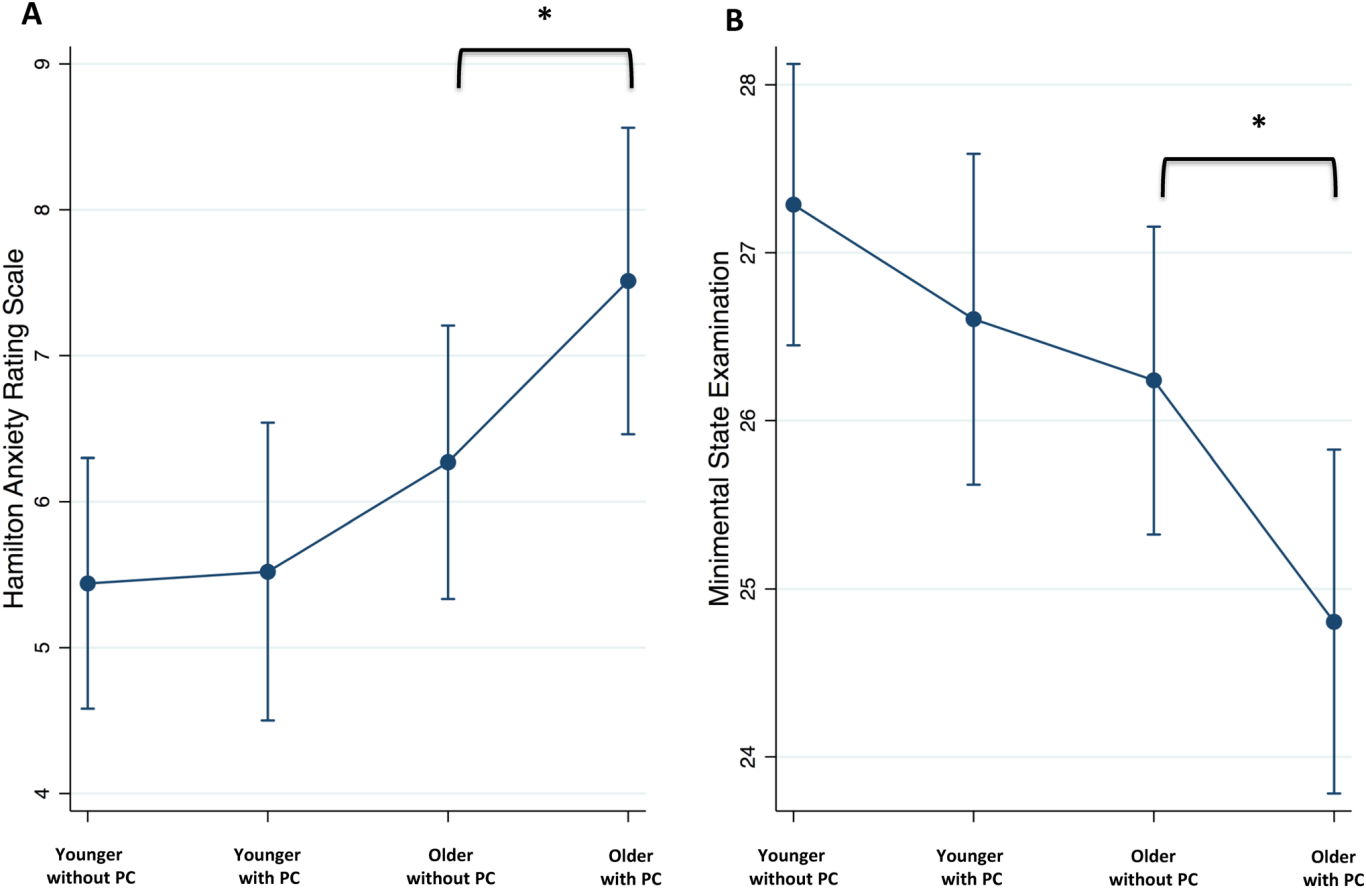
Age dependence of predictive values of prodromal constipation (PC) for higher Hamilton Depression scale (HAM) (**a**) and lower Mini-mental State Examination (MMSE) (**b**). Error bars indicate 95% confidence intervals. Asterisks indicate significant interaction effect in linear regression models (*p* < 0.05)

Prevalence of PC was 38.7% in younger and 40.4% in older patients (*p* = 0.084). In older patients, PC was associated with greater odds of being treated with L-dopa (OR: 11.58, 95%CI: 1.85–72.40, *p* = 0.009) and complaining about apathy (OR: 7.34, 95%CI: 1.79–30.07, *p* = 0.006). There was also a trend towards significance for greater odds of attention/memory complaints (OR: 3.46, 95%CI: 0.96–12.49, *p* = 0.057) and a significant association with lower MMSE (coeff.: − 2.48, 95%CI: − 4.00 to − 0.96, *p* = 0.001) and higher HAM (coeff.: 2.07, 95%CI: 0.51–3.62, *p* = 0.009) (Fig. 1a, b). Conversely in younger patients, no significant association was detected (Table 1).

## Discussion

In the present prospective longitudinal study, we explored the association between the presence of PC and disease phenotype in the early stages considering the impact of sex and age in a large cohort of PD patients.

In line with previous evidence, our cohort presented an overall prevalence of PC of approximately 40%, with higher frequency in women and older patients [4, 15]. When examining the impact of sex, the presence of PC was associated with attention/memory complaints and apathy as well as with a trend towards significance for lower cognitive performances in women only. Complementary, when considering the impact of age, the presence of PC was associated with attention/memory complaints and apathy, higher rates of treatment with levodopa as well as with lower cognitive performances and more severe apathy in older patients only (Fig. 1a, b).

Previous evidence showed high sex- and age-related heterogeneity of several prodromal markers of PD supporting the notion that such demographic factors should be taken into account when evaluating tools or algorithms for PD prediction [2–4]. Accordingly, PC confers greater risk of developing PD in women and healthy subjects aged above 65 year-old [4]. On the other hand and similarly to other autonomic symptoms, once the motor symptoms have manifested, the presence of constipation since the early phase represents a risk factor for a more severe motor and cognitive burden of disease [16–18]. Notwithstanding, to date, evidence suggesting a relationship between the presence of constipation in prodromal phase and disease phenotype after onset of motor symptoms are lacking. Here, we demonstrated that PC might anticipate a specific phenotype of the disease characterized by early involvement of cognitive and behavioural domains especially in women and older patients. Also, irrespective of sex, older patients reporting PC have higher odds of receiving levodopa reflecting a greater motor burden of disease.

Also in line with the hypothesis of a gut-brain axis, the role of PC as a prodromal symptom for PD seems to confirm the Braak’s model for the progression of Lewy pathology, with an early involvement of the enteric nervous system and dorsal motor nucleus of the vagus even before degeneration of substantia nigra begins [8, 9, 16]. Accordingly, PC is considered one of the earliest marker of autonomic dysfunction in PD. As such our findings are in line with previous data suggesting that early autonomic dysfunction has to be considered as a risk marker for a more severe form of disease since the earliest stages [13, 16–19]. Recent evidence suggests several risk and prodromal markers of PD (including constipation, male sex and age) are associate with diverse gut microbiome composition further supporting the pivotal role of the gut-brain axis in disease pathogenesis [20]. Our data demonstrate for the first time (1) the association between PC and early disease phenotype as well as (2) the sex- and age-dependency of such relationship.

We acknowledge our study has limitations. First, the lack of a longer longitudinal phase precludes the possibility of describing the complete natural history of PD patients with PC. Notwithstanding, this is the largest study to date evaluating prospectively the sex- and age-related relationship between PC and both motor and non-motor symptoms in early PD. Second, as the presence of prodromal constipation was annotated with a patient interview, we recognize the possibility of a recall bias. Similarly the majority of non-motor symptoms were evaluated with a semistructured interview and not with objective testing. However, global cognition and depression were evaluated with validated scales. Although we recognize the sample drop at follow-up as a further limitation, mixed-effect regression model should handle missing data to some extent. Finally, the lack of a control group from the general population represents another drawback as constipation is highly frequent in the general population, especially in women and older subjects. Thus, we cannot exclude such findings are detected also in healthy controls. A large body of evidence show the influence of sex and age on both prevalence as well as pattern of constipation symptoms [21]. Future studies should investigate the impact of demographic features on specific symptoms of constipation in PD compared to general population. Also, recent evidence suggests a complex interaction between microbial composition and risk and prodromal markers of PD with sex and age being associated with different microbial measures [20]. Thus, we are aware we are describing only part of a manifold framework. Notwithstanding, this is the first study attempting at describing the impact of sex and age on the relationship between PC and phenotype of disease.

In conclusion, we demonstrated that PC anticipates lower cognitive performances and more severe apathy since the earliest stages of PD in women and older patients. In addition, older patients reporting PC might present a greater motor burden of disease. Sex- and age-related heterogeneity of prodromal markers of PD may have an impact on disease phenotype.

### Electronic supplementary material

Below is the link to the electronic supplementary material.

Supplementary material 1 (DOCX 78 kb)

## Data Availability

All data are available from the corresponding authors upon request.

## Appendix: The PRIAMO study group

**Steering committee**

Angelo Antonini, University of Padua; Paolo Barone, University of Salerno; Carlo Colosimo, Department of Neurology, Santa Maria University Hospital; Roberto Marconi, Ospedale della Misericordia, Grosseto; Letterio Morgante, Dipartimento di Neuroscienze, Scienze Psichiatriche ed Anestesiologiche, Universita` di Messina, Italy.

**Participating centers**

Benincasa D, Ospedale Sant’Andrea II Fac. Med. Chir., Universita` La Sapienza, Roma; Quatrale R, Biguzzi S, Ospedale Sant’Anna,; Braga M, Ospedale Civile, Vimercate; Ceravolo G, Capecci M, Ospedale Umberto I, Ancona; Meco G, Caravona N, Universita`La Sapienza, Roma; Scala R, De Falco FA, Ospedale S. Maria Loreto Nuovo, Napoli; Pezzoli G, De Gaspari D, Parkinson Institute, ICP, Milano; Bottacchi E, Di Giovanni M, Ospedale Regionale, Aosta; Cannas A, Floris G, Clinica Neurologica, Policlinico Universatario, Monserrato, Cagliari; Gallerini S, Grasso L, Ospedale della Misericordia, Grosseto; Gaglio RM, Gurgone G, Azienda Ospedaliera S.Giovanni di Dio, Agrigento; Volpe G, AOU S. Giovanni di Dio e Ruggi d’Aragona, Salerno; Zappulla S, Neurologia, Ospedale Umberto I, Enna; Ceravolo R, Kiferle L, Ospedale Santa Chiara, Pisa; Ramat S, Meoni S, Clinica Neurologica, Azienda Ospedaliera Universitaria,; Pisani A, Moschella V, Policlinico Tor Vergata, Roma; Morgante F, Savica R, Dipartimento di Neuroscienze, Scienze Psichiatriche ed Anestesiologiche, Universita` of Messina; Pepe F, Fondazione Poliambulanza, Brescia; Ciccarelli G, Petretta V, A.O.R.N. San Giuseppe Moscati,; Giglia RM, Randisi MG, Azienda Ospedaliera S. Elia, Caltanissetta; Iemolo F, U.O. Neurologia, ospedale Guzzardi, Ragusa; Avarello TP, Romeno M, Ospedale Villa Sofia CTO, Palermo; Santangelo G, Universita` Federico II, Napoli; Stocchi F, IRCCS San Raffaele, Roma; Sciortino G, P.O. Guzzardi, Vittoria; Sorbello V, Policlinico Universitario, Nicoletti A,; Tiple D, Fabbrini G, Universita` La Sapienza, Roma; Bentivoglio A, Pontieri FE, Guidubaldi A, Universita` Cattolica S. Cuore, Roma; Muoio R, Neuromed IRCCS, Pozzilli; Toni V, P.O. F. Ferrari, Casarano; Del Dotto P, Logi C, Ospedale Versilia, Camaiore; Ciacci G, Ulivelli M, Policlinico Le Scotte, Siena; Perini M, Lanfranchi S, Ospedale S. Antonio Abate, Gallarate; Griffini S, Troianiello B, Istituto Clinico Citta` di Brescia; Baratti M, Amidei S, Ospedale Ramazzini, Carpi; Consoli D, Iellamo M, Ospedale G. Iazzolino, Vibo Valentia; Cuomo T, Ospedale Civile Umberto I, Nocera Inferiore; Scaglioni A, Medici D, Ospedale di Vaio, Fidenza; Manfredi M, Fondazione Salvatore Maugeri IRCCS, Castel Goffredo, Mantova; Abbruzzese G, Di Brigida G, Universita` degli Studi di Genova, Genova; Cocco GA, Agnetti V, Universita` degli Studi di Sassari, Sassari; Cossu G, Deriu M, Azienda Ospedaliera G. Brotzu, Cagliari; Abrignani M, Modica C, Ospedale San Biagio di Marsala, Marsala; Albani G, Milan E, Istituto Scientifico San Giuseppe, Piancavallo; Martinelli P, Scaglione C, Universita` di Bologna, Bologna; Mucchiut M, Zanini S, Policlinico Universitario, Udine; Pennisi F, Ospedale di Castelvetrano, Castelvetrano, Trapani; Soliveri P, Albanese A, Istituto Nazionale Neurologico C. Besta, Milano; Pederzoli Massimo, Neurologia, Ospedale Civile, Vimercate, Milano; Bartolomei L, L’erario R, Ospedale Civile San Bortolo, Vicenza; Capus L, Ferigo L, Ospedale di Gattinara, Trieste; Marano R, Nastasi V, Azienda Ospedaliera Papardo, Messina; Luciano R, Maiello L, Ospedale Monaldi, Napoli; Simone P, Fogli D, Ospedale Casa Sollievo della Sofferenza, San Giovanni Rotondo; Lopiano L, Pesare M, A.S.O. Molinette, Torino; Nordera G, Pilleri E, Casa di Cura Villa Margherita, Arcugnano; Scaravilli T, Azienda Ospedaliera Padova, Padova; Giaccaglini E, Alesi C, Ospedale Civile, Jesi, Italy; Petrone A, Neurologia, Presidio Ospedaliero Annunziata, Cosenza; Trianni G, Neurologia, P.O.F. Ferrari, Casarano, Lecce.

## Abbreviations

ANOVA: Analysis of variance
EQ-VAS: EuroQol visual analogue scale
HAM: Hamilton depression scale
HRQol: Health-related quality of life
H&Y: Hoehn and Yahr
LR: Likelihood ratio
MDS: The movement disorder society
MMSE: Mini Mental State examination
NMS: Non-motor symptoms
OR: Odds ratio
PC: Prodromal constipation
PD: Parkinson’s disease
PDQ-39: 39 items Parkinson’s disease questionnaire
PRIAMO: PaRkinson dIseAse non-MOtor symptoms
SD: Standard deviation
UPDRS-III: Unified Parkinson’s Disease Rating Scale part III

## References

[1] Heizel S, Berg D, Gasser T, Chen H, Yao C, Postuma RB, MDS Task Force on the Definition of Parkinson’s Disease (2019). Update of the MDS Research criteria for prodromal Parkinson’s disease. Mov Disord.

[2] Pilotto A, Heinzel S, Suenkel U, Lerche S, Brockmann K, Roeben B, Schaeffer E, Wurster I, Yilmaz R, Liepelt-Scarfone I, von Thaler AK, Metzger FG, Eschweiler GW, Postuma RB, Maetzler W, Berg D (2017). Application of the Movement Disorder Society prodromal Parkinson’s disease research criteria in 2 independent prospective cohorts. Mov Disord.

[3] Schrag A, Anastasiou Z, Ambler G, Noyce A, Walters K (2020). Predicting diagnosis of Parkinson’s disease: a risk algorithm based on primary care presentations. Mov Disord.

[4] Heinzel S, Kasten M, Behnke S, Vollstedt EJ, Klein C, Hagenah J, Pausch C, Heilmann R, Brockmann K, Suenkel U, Yilmaz R, Liepelt-Scarfone I, Walter U, Berg D (2018). Age- and sex-related heterogeneity in prodromal Parkinson’s disease. Mov Disord.

[5] Cottone C, Tosetti C, Disclafani G, Ubaldi E, Cogliandro R, Stanghellini V (2014). Clinical features of constipation in general practice in Italy. United Eur Gatroenterol J.

[6] Yu Q, Yu S, Zuo L, Lian T, Hu Y, Wang R, Piano Y, Guo P, Liu L, Jin Z, Li L, Chan P, Chen S, Wang X, Zhang W (2018). Parkinson disease with constipation: clinical features and relevant factors. Sci Rep.

[7] Martinez-Martin P, Pecurariu CF, Odin P, van Hilten J, Antonini A, Rojo-Abuin JM, Borges V, Trenkwalder C, Aarsland D, Brooks DJ, Chaudhuri KR (2012). Gender-related differences in the burden of non-motor symptoms in Parkinson’s disease. J Neurol.

[8] Stokholm MG, Danielsen EH, Hamilton-Dutoit SJ, Borghammer P (2016). Pathological α-synuclein in gastrointestinal tissues from prodromal Parkinson disease patients. Ann Neurol.

[9] Breen DP, Halliday GM, Lang AE (2019). Gut–brain axis and the spread of α-synuclein pathology: vagal highway or dead end?. Mov Disord.

[10] Antonini A, Colosimo C, Marconi R, Morgante L, Barone P, PRIAMO study group (2008). The PRIAMO study: background, methods and recruitment. Neurol Sci.

[11] Barone P, Antonini A, Colosimo C, Marconi R, Morgante L, Avarello TP, Bottacchi E, Cannas A, Ceravolo G, Ceravolo R, Cicarelli G, Gaglio RM, Giglia RM, Iemolo F, Manfredi M, Meco G, Nicoletti A, Pederzoli M, Petrone A, Pisani A, Pontieri FE, Quatrale R, Ramat S, Scala R, Volpe G, Zappulla S, Bentivoglio AR, Stocchi F, Trianni G, Dotto PD, PRIAMO study group (2009). The PRIAMO study: a multicenter assessment of nonmotor symptoms and their impact on quality of life in Parkinson’s disease. Mov Disord.

[12] Antonini A, Barone P, Marconi R, Morgante L, Zappulla S, Pontieri FE, Ramat S, Ceravolo MG, Meco G, Cicarelli G, Pederzoli M, Manfredi M, Ceravolo R, Mucchiut M, Volpe G, Abbruzzese G, Bottacchi E, Bartolomei L, Ciacci G, Cannas A, Randisi MG, Petrone A, Baratti M, Toni V, Cossu G, Del Dotto P, Bentivoglio AR, Abrignani M, Scala R, Pennisi F, Quatrale R, Gaglio RM, Nicoletti A, Perini M, Avarello T, Pisani A, Scaglioni A, Martinelli PE, Iemolo F, Ferigo L, Simone P, Soliveri P, Troianiello B, Consoli D, Mauro A, Lopiano L, Nastasi G, Colosimo C (2012). The progression of non-motor symptoms in Parkinson’s disease and their contribution to motor disability and quality of life. J Neurol.

[13] Picillo M, Palladino R, Barone P, Erro R, Colosimo C, Marconi R, Morgante L, Antonini A, PRIAMO Study Group (2017). The PRIAMO study: urinary dysfunction as a marker of disease progression in early Parkinson’s disease. Eur J Neurol.

[14] Picillo M, Palladino R, Erro R, Colosimo C, Marconi R, Antonini A, Barone P, PRIAMO study group (2019). The PRIAMO study: active sexual life is associated with better motor and non-motor outcomes in men with early Parkinson’s disease. Eur J Neurol.

[15] Savica R, Carlin JM, Grossardt BR, Bower JH, Ahlskog JE, Maraganore DM, Bharucha AE, Rocca WA (2009). Medical records documentation of constipation preceding Parkinson disease: a case-control study. Neurology.

[16] De Pablo-Fernandez E, Tur C, Revesz T, Lees AJ, Holton JL, Warner TT (2017). Association of autonomic dysfunction with disease progression and survival in Parkinson Disease. JAMA Neurol.

[17] Jones JD, Rahmani E, Garcia E, Jacobs JP (2020). Gastrointestinal symptoms are predictive of trajectories of cognitive functioning in de novo Parkinson’s disease. Parkinson Relat Disord.

[18] Liping Z, Qiying S, Jifeng G, Xinxiang Y, Beisha T (2018). Study on the clinical features and related factors of constipation in patients with parkinson’s disease. J Chin Phys.

[19] Sauerbier A, Jenner P, Todorova A, Chaudhuri KR (2016). Non motor subtypes and Parkinson’s disease. Parkinson Relat Disord.

[20] Heinzel S, Aho VTE, Suenkel U, von Thaler AK, Schulte C, Deuschle C, Paulin L, Hantunen S, Brockmann K, Eschweiler GW, Maetzler W, Berg D, Auvinen P, Scheperjans F (2020). Gut microbiome signatures of risk and prodromal markers of Parkinson disease. Ann Neurol.

[21] Verkujl SJ, Meinds RJ, Trzpis M, Broens PMA (2020). The influence of demographic characteristics on constipation symptoms: a detailed overview. BMC Gastroenterol.

