# Supplementary material for: The PRIAMO study: age- and sex-related relationship between prodromal constipation and disease phenotype in early Parkinson’s disease
Source: J Neurol. 2020 Aug 18;268(2):448–54. doi: 10.1007/s00415-020-10156-3 (PMC7880965; doi:10.1007/s00415-020-10156-3)
Supplement: Supplementary file 1 — Supplementary material 1 (DOCX 78 kb) [file 415_2020_10156_MOESM1_ESM.docx]

**Supplemental material**

**PRIAMO semistructured interview exploring NMS domains (dichotomus answers, yes/no)**

Patients were asked to report each specific symptom as affected/not affected with reference to the month before the visit. Both patients and their caregivers contributed to the completion of the interview.

1)Does the patient have the following gastrointestinal disturbances?

-drooling

-swallowing difficulties

-nausea/vomit

-constipation

-anorectal dysfunction

-incomplete bowel emptying

-fecal incontinence

2)Does the patient have the following pain symptoms?

-undefinied pain

-lower limbs pain

-abdominal pain

-pain (after assumption of antiparkinsonian medications)

-shoulder pain

3)Does the patient have the following urinary symptoms?

-urgency

-frequency (need of voiding every 2 hours or less)

-nocturia

4)Does the patient have the following cardiovascular symptoms?

-dizziness, lightheadness during postural changes

-falls after syncope

5)Does the patient have the following sleep disturbances?

-RBD (talking or moving while sleeping)

-difficulties in falling asleep

-diurnal somnolence

-restless legs syndrome

6)Does the patient have fatigue severe enough to limit activities of daily living?

7)Does the patient have apathy?

-lost of interest for sorroundings, activities of daily living or new activities

8)Does the patient have impared attention/memory?

-difficulties in sustaining attention

-forgetful for recent events/talks or activities of daily living

9)Does the patient have the following skin problems?

-seborrhea

-hyperhidrosis

10)Does the patient have the following psychiatric disturbances?

-anhedonia

-anxiety

-panic attacks

-aggressiveness

-suicide ideas

-irritability

-unexplained worry

-sadness/depression

-psychotic thinking

-hallucinations

11)Does the patient have the following respiratory disturbances?

-dyspnoea

-stridor

-cough

12)Does the patient have the following other non motor symptoms?

-smell disturbances

-taste disturbances

-weight changes

-diplopia

-sexual disturbances

**Supplemental table 1. Demographic and clinical characteristics of Parkinson’s disease patients with (PD-C) and**

**without prodromal constipation (PD-NC) by sex at each time point (T1, T2 and T3)**

|  | **Men (N = 247)** | |  | **Women (N = 138)** | |  | |
| --- | --- | --- | --- | --- | --- | --- | --- |
|  | **PD-NC** | **PD-C** | **p** | **PD-NC** | **PD-C** | **p** | |
| **T1** | | | | | | |  |
| Age, years | 65.05 (9.57) | 68.24 (8.96) | **0.012** | 65.83 (8.82) | 67.92 (7.57) | 0.141 | |
| Age at onset, years | 59.98 (10.87) | 63.13 (10.40) | **0.030** | 59.95 (11.19) | 62.76 (7.94) | 0.096 | |
| Disease duration, years | 4.84 (3.75) | 5.18 (4.41) | 0.534 | 5.88 (7.55) | 5.16 (4.54) | 0.508 | |
| UPDRS-III | 19.52 (10.26) | 18.93 (9.71) | 0.661 | 19.91 (10.00) | 19.27 (9.24) | 0.699 | |
| HAM | 6.41 (4.87) | 6.62 (4.50) | 0.739 | 6.37 (4.60) | 7.85 (5.92) | 0.105 | |
| EQ-VAS | 73.68 (16.16) | 68.79 (20.52) | **0.042** | 65.38 (19.87) | 67.72 (22.70) | 0.521 | |
| PDQ-39 | 154.27 (97.40) | 179.30 (126.66) | 0.146 | 194.03 (102.92) | 220.90 (139.24) | 0.367 | |
| MMSE | 27.48 (3.27) | 26.20 (5.92) | **0.029** | 27.27 (2.54) | 25.75 (6.49) | 0.064 | |
| L-dopa N (%) | 94 (57.7) | 51 (60.7) | 0.746 | 50 (66.7) | 36 (57.1) | 0.330 | |
| Urinary, N (%) | 91 (55.8) | 51 (60.7) | 0.548 | 31 (41.3) | 29 (46.0) | 0.702 | |
| Pain, N (%) | 88 (54.0) | 47 (56.0) | 0.874 | 49 (65.3) | 40 (63.5) | 0.963 | |
| Cardiovascular, N (%) | 18 (11.0) | 14 (16.7) | 0.295 | 11 (14.7) | 13 (20.6) | 0.486 | |
| Sleep, N (%) | 101 (62.0) | 51 (60.7) | 0.958 | 41 (54.7) | 39 (61.9) | 0.493 | |
| Fatigue, N (%) | 72 (44.2) | 40 (47.6) | 0.703 | 52 (69.3) | 40 (63.5) | 0.587 | |
| Apathy, N (%) | 44 (27.0) | 27 (32.1) | 0.485 | 19 (25.3) | 25 (39.7) | 0.106 | |
| Attention/memory, N (%) | 68 (41.7) | 40 (47.6) | 0.453 | 23 (30.7) | 34 (54.0) | **0.009** | |
| Skin, N (%) | 34 (20.9) | 18 (21.4) | 1.000 | 4 (5.3) | 6 (9.5) | 0.538 | |
| Psychiatric, N (%) | 95 (58.3) | 54 (64.3) | 0.438 | 46 (61.3) | 43 (68.3) | 0.504 | |
| Respiratory, N (%) | 25 (15.3) | 14 (16.7) | 0.930 | 16 (21.3) | 9 (14.3) | 0.396 | |
| Other symptoms, N (%) | 88 (54.0) | 36 (42.9) | 0.128 | 35 (46.7) | 31 (49.2) | 0.899 | |
| **T2** | | | | | | |  |
| Age, years | 65.80 (8.99) | 68.92 (9.97) | 0.133 | 66.82 (8.49) | 68.45 (7.92) | **0.017** | |
| UPDRS-III | 20.18 (10.21) | 22.10 (12.71) | 0.253 | 18.88 (9.51) | 21.20 (11.05) | 0.216 | |
| HAM | 5.07 (4.37) | 5.77 (3.96) | 0.289 | 6.02 (4.44) | 7.29 (5.17) | 0.152 | |
| EQ-VAS | 72.12 (19.10) | 69.23 (20.01) | 0.326 | 66.76 (17.45) | 65.57 (23.94) | 0.754 | |
| PDQ-39 | 159.78 (111.81) | 175.63 (100.56) | 0.419 | 184.31 (117.53) | 183.50 (124.55) | 0.978 | |
| MMSE | 27.01 (4.28) | 24.84 (7.18) | **0.008** | 27.09 (5.29) | 26.82 (3.54) | 0.745 | |
| L-dopa, N (%) | 96 (70.1) | 46 (71.9) | 0.924 | 52 (80.0) | 40 (71.4) | 0.375 | |
| Urinary, N (%) | 81 (59.1) | 45 (70.3) | 0.170 | 30 (46.2) | 32 (57.1) | 0.306 | |
| Pain, N (%) | 76 (55.5) | 33 (51.6) | 0.714 | 50 (76.9) | 37 (66.1) | 0.262 | |
| Cardiovascular, N (%) | 16 (11.7) | 13 (20.3) | 0.159 | 15 (23.1) | 8 (14.3) | 0.319 | |
| Sleep, N (%) | 96 (70.1) | 41 (64.1) | 0.490 | 36 (55.4) | 32 (57.1) | 0.992 | |
| Fatigue, N (%) | 67 (48.9) | 30 (46.9) | 0.907 | 44 (67.7) | 34 (60.7) | 0.542 | |
| Apathy, N (%) | 39 (28.5) | 22 (34.4) | 0.494 | 17 (26.2) | 17 (30.4) | 0.756 | |
| Attention/memory, N (%) | 62 (45.3) | 34 (53.1) | 0.374 | 23 (35.4) | 21 (37.5) | 0.959 | |
| Skin, N (%) | 34 (24.8) | 14 (21.9) | 0.781 | 11 (16.9) | 6 (10.7) | 0.473 | |
| Psychiatric, N (%) | 73 (53.3) | 36 (56.2) | 0.809 | 43 (66.2) | 35 (62.5) | 0.819 | |
| Respiratory, N (%) | 24 (17.5) | 14 (21.9) | 0.588 | 12 (18.5) | 7 (12.5) | 0.517 | |
| Other symptoms, N (%) | 82 (59.9) | 29 (45.3) | 0.075 | 26 (40.0) | 29 (51.8) | 0.265 | |
| **T3** | | | | | | |  |
| Age, years | 65.98 (9.67) | 69.41 (9.84) | 0.100 | 67.18 (8.17) | 68.32 (8.18) | 0.190 | |
| UPDRS-III | 22.99 (10.70) | 23.73 (12.41) | 0.664 | 20.73 (11.50) | 24.18 (15.42) | 0.206 | |
| HAM | 5.08 (4.16) | 5.72 (4.42) | 0.325 | 5.49 (4.93) | 7.22 (6.78) | 0.156 | |
| EQ-VAS | 70.94 (21.17) | 68.39 (20.71) | 0.423 | 62.01 (20.07) | 64.21 (27.67) | 0.649 | |
| PDQ-39 | 161.86 (99.69) | 186.09 (115.99) | 0.211 | 167.12 (107.29) | 187.00 (147.17) | 0.608 | |
| MMSE | 25.68 (7.21) | 26.53 (4.45) | 0.384 | 25.92 (7.32) | 23.92 (8.73) | 0.216 | |
| L-dopa, N (%) | 102 (79.1) | 55 (83.3) | 0.603 | 39 (79.6) | 38 (71.7) | 0.487 | |
| Urinary, N (%) | 78 (60.5) | 41 (62.1) | 0.945 | 24 (49.0) | 28 (52.8) | 0.849 | |
| Pain, N (%) | 70 (54.3) | 36 (54.5) | 1 | 39 (79.6) | 32 (60.4) | 0.058 | |
| Cardiovascular, N (%) | 9 (7.0) | 9 (13.6) | 0.215 | 5 (10.2) | 4 (7.5) | 0.902 | |
| Sleep, N (%) | 86 (66.7) | 45 (68.2) | 0.958 | 27 (55.1) | 35 (66.0) | 0.354 | |
| Fatigue, N (%) | 69 (53.5) | 37 (56.1) | 0.850 | 29 (59.2) | 31 (58.5) | 1 | |
| Apathy, N (%) | 31 (24.0) | 26 (39.4) | **0.039** | 7 (14.3) | 18 (34.0) | **0.038** | |
| Attention/memory, N (%) | 50 (38.8) | 37 (56.1) | **0.032** | 18 (36.7) | 26 (49.1) | 0.291 | |
| Skin, N (%) | 34 (26.4) | 10 (15.2) | 0.112 | 6 (12.2) | 5 (9.4) | 0.890 | |
| Psychiatric, N (%) | 73 (56.6) | 39 (59.1) | 0.856 | 27 (55.1) | 29 (54.7) | 1 | |
| Respiratory, N (%) | 20 (15.6) | 12 (18.2) | 0.802 | 6 (12.2) | 5 (9.4) | 0.890 | |
| Other symptoms, N (%) | 85 (65.9) | 32 (49.2) | **0.037** | 21 (42.9) | 26 (49.1) | 0.668 | |

Counts (percentages) and means (standard deviations) are reported for categorical and continuous variables, respectively.

Abbreviations: EQ-VAS: EuroQol visual analogue scale; HAM: the Hamilton depression scale; MMSE: Mini-mental state examination;

PD-C= Parkinson’s disease with prodromal constipation; PD-NC= Parkinson’s disease without prodromal constipation; PDQ-39:

39-item Parkinson’s disease questionnaire; UPDRS-III: Unified Parkinson’s disease rating scale part III.

Significant results are highlighted in bold.

**Supplemental Table 2. Impact of age considered as a continuous variable on the relationship between prodromal constipation and motor and non motor symptoms in PD.**

| **Continuous variables** | **Estimate** | **LCI** | **UCI** | **p** |
| --- | --- | --- | --- | --- |
| **UPDRS-III** | 0.16 | -0.0002 | 0.322 | 0.050 |
| **MMSE** | -0.02 | -0.09 | -0.04 | 0.457 |
| **EQ-VAS** | -0.06 | -0.35 | -0.22 | 0.668 |
| **PDQ-39** | -0.11 | -1.99 | 1.77 | 0.908 |
| **HAM** | -0.06 | -0.13 | 0.01 | 0.093 |
| **Categorical variables** | **OR** | **LCI** | **UCI** | **p** |
| **L-dopa treatment** | 0.98 | 0.97 | 1.11 | 0.226 |
| **Urinary** | 1.04 | 0.52 | 3.05 | 0.601 |
| **Pain** | 0.98 | 0.92 | 1.03 | 0.477 |
| **Cardiovascular** | 1.03 | 0.96 | 1.11 | 0.287 |
| **Sleep** | 1.00 | 0.94 | 1.06 | 0.911 |
| **Fatigue** | 0.97 | 0.92 | 1.03 | 0.399 |
| **Apathy** | 0.97 | 0.91 | 1.03 | 0.378 |
| **Attention/**  **memory** | 1.05 | 0.99 | 1.11 | 0.077 |
| **Skin** | 0.96 | 0.90 | 1.02 | 0.272 |
| **Psychiatric** | 0.96 | 0.91 | 1.02 | 0.234 |
| **Respiratory** | 1.06 | 1.00 | 1.13 | **0.049** |
| **Other symptoms** | 0.95 | 0.88 | 1.02 | 0.174 |

Coefficients are presented as adjusted estimates (for continuous dependent variables) or odds ratios (for categorical dependent variables) along with 95% confidence intervals (95%CI) and were estimated using linear (for continuous dependent variables) and logistic (for categorical dependent variables) mixed effects regression models, adjusting for disease duration and time point, introducing a random effect for participant. Significant results are highlighted in bold.

Abbreviations: EQ-VAS: EuroQol visual analogue scale; HAM: the Hamilton depression scale; LCI= lower 95% confidence interval; MMSE= Mini-mental state examination; OR= odds ratio; PD= Parkinson’s disease; PDQ-39: 39-item Parkinson’s disease questionnaire; UCI: upper 95% confidence interval; UPDRS-III: Unified Parkinson’s disease rating scale part III.

**APPENDIX: THE PRIAMO STUDY GROUP**

**Steering Committee**

Angelo Antonini, University of Padua; Paolo Barone, University of Salerno; Carlo Colosimo, Department of Neurology, Santa Maria University Hospital; Roberto Marconi, Ospedale della Misericordia, Grosseto; Letterio Morgante, Dipartimento di Neuroscienze, Scienze Psichiatriche ed Anestesiologiche, Universita` di Messina, Italy.

**Participating Centers**

Benincasa D, Ospedale Sant’Andrea II Fac. Med. Chir., Universita` La Sapienza, Roma; Quatrale R, Biguzzi S, Ospedale Sant’Anna,; Braga M, Ospedale Civile, Vimercate; Ceravolo G, Capecci M, Ospedale Umberto I, Ancona; Meco G, Caravona N, Universita`La Sapienza, Roma; Scala R, De Falco FA, Ospedale S. Maria Loreto Nuovo, Napoli; Pezzoli G, De Gaspari D, Parkinson Institute, ICP, Milano; Bottacchi E, Di Giovanni M, Ospedale Regionale, Aosta; Cannas A, Floris G, Clinica Neurologica, Policlinico Universatario, Monserrato, Cagliari; Gallerini S, Grasso L, Ospedale della Misericordia, Grosseto; Gaglio RM, Gurgone G, Azienda Ospedaliera S.Giovanni di Dio, Agrigento; Volpe G, AOU S. Giovanni di Dio e Ruggi d’Aragona, Salerno; Zappulla S, Neurologia, Ospedale Umberto I, Enna; Ceravolo R, Kiferle L, Ospedale Santa Chiara, Pisa; Ramat S, Meoni S, Clinica Neurologica, Azienda Ospedaliera Universitaria,; Pisani A, Moschella V, Policlinico Tor Vergata, Roma; Morgante F, Savica R, Dipartimento di Neuroscienze, Scienze Psichiatriche ed Anestesiologiche, Universita` of Messina; Pepe F, Fondazione Poliambulanza, Brescia; Ciccarelli G, Petretta V, A.O.R.N. San Giuseppe Moscati, ; Giglia RM, Randisi MG, Azienda Ospedaliera S. Elia, Caltanissetta; Iemolo F, U.O. Neurologia, ospedale Guzzardi, Ragusa; Avarello TP, Romeno M, Ospedale Villa Sofia CTO, Palermo; Santangelo G, Universita` Federico II, Napoli; Stocchi F, IRCCS San Raffaele, Roma; Sciortino G, P.O. Guzzardi, Vittoria; Sorbello V, Policlinico Universitario, Nicoletti A, ; Tiple D, Fabbrini G, Universita` La Sapienza, Roma; Bentivoglio A, Pontieri FE, Guidubaldi A, Universita` Cattolica S. Cuore, Roma; Muoio R, Neuromed IRCCS, Pozzilli; Toni V, P.O. F. Ferrari, Casarano; Del Dotto P, Logi C, Ospedale Versilia, Camaiore; Ciacci G, Ulivelli M, Policlinico Le Scotte, Siena; Perini M, Lanfranchi S, Ospedale S. Antonio Abate, Gallarate; Griffini S, Troianiello B, Istituto Clinico Citta` di Brescia; Baratti M, Amidei S, Ospedale Ramazzini, Carpi; Consoli D, Iellamo M, Ospedale G. Iazzolino, Vibo Valentia; Cuomo T, Ospedale Civile Umberto I, Nocera Inferiore; Scaglioni A, Medici D, Ospedale di Vaio, Fidenza; Manfredi M, Fondazione Salvatore Maugeri IRCCS, Castel Goffredo, Mantova; Abbruzzese G, Di Brigida G, Universita` degli Studi di Genova, Genova; Cocco GA, Agnetti V, Universita` degli Studi di Sassari, Sassari; Cossu G, Deriu M, Azienda Ospedaliera G. Brotzu, Cagliari; Abrignani M, Modica C, Ospedale San Biagio di Marsala, Marsala; Albani G, Milan E, Istituto Scientifico San Giuseppe, Piancavallo; Martinelli P, Scaglione C, Universita` di Bologna, Bologna; Mucchiut M, Zanini S, Policlinico Universitario, Udine; Pennisi F, Ospedale di Castelvetrano, Castelvetrano, Trapani; Soliveri P, Albanese A, Istituto Nazionale Neurologico C. Besta, Milano; Pederzoli Massimo, Neurologia, Ospedale Civile, Vimercate, Milano; Bartolomei L, L’erario R, Ospedale Civile San Bortolo, Vicenza; Capus L, Ferigo L, Ospedale di Gattinara, Trieste; Marano R, Nastasi V, Azienda Ospedaliera Papardo, Messina; Luciano R, Maiello L, Ospedale Monaldi, Napoli; Simone P, Fogli D, Ospedale Casa Sollievo della Sofferenza, San Giovanni Rotondo; Lopiano L, Pesare M, A.S.O. Molinette, Torino; Nordera G, Pilleri E, Casa di Cura Villa Margherita, Arcugnano; Scaravilli T, Azienda Ospedaliera Padova, Padova; Giaccaglini E, Alesi C, Ospedale Civile, Jesi, Italy; Petrone A, Neurologia, Presidio Ospedaliero Annunziata, Cosenza; Trianni G, Neurologia, P.O.F. Ferrari, Casarano, Lecce.
